# Using field notes to evaluate competencies in family medicine training: a study of predictors of intention

**Published:** 2013-03-31

**Authors:** Miriam Lacasse, Frédéric Douville, Émilie Desrosiers, Luc Côté, Stéphane Turcotte, France Légaré

**Affiliations:** 1 Département de médecine familiale et de médecine d’urgence, Faculté de médecine, Université Laval, Quebec City, Canada; 2 Faculté des sciences infirmières, Université Laval, Quebec City, Canada; 3 Faculté de médecine at Université Laval, Quebec City, Canada; 4 Knowledge Transfer and Health Technology Assessment Research Group, Centre de recherche du CHUQ, Hôpital Saint-François d’Assise, Quebec City, Canada

## Abstract

**Background:**

Documenting feedback during clinical supervision using field notes (FN) is a recommended competency-based evaluation strategy that will require changes in the culture of medical education. This study identified factors influencing the intention to adopt FN in family medicine training, using the theory of planned behaviour.

**Methods:**

This mixed-methods study involved clinical teachers (CT) and residents from two family medicine units. Main outcomes were: 1) intention (and its predictors: attitude, perceived behavioural control (PBC) and normative belief) to use FN, assessed using a 7-item Likert scale questionnaire (1: *strongly disagree* to 7: *strongly agree*) and 2) related salient beliefs, explored in focus groups three and six months after FN implementation.

**Results:**

27 CT and 28 residents participated. Intention to use FN was 6.20±1.20 and 5.74±1.03 in CT and residents respectively. Predictors of this intention were attitude and PBC (mutually influential: *p <* 0.05), and normative belief (*p <* 0.01). Focus groups identified underlying beliefs regarding their use (perceived advantages/disadvantages and facilitators/barriers).

**Conclusion:**

Intention to adopt field notes to document competency is influenced by attitude, perceived behavioural control and normative belief. Implementation of field notes should be preceded by interventions that target the identified salient beliefs to improve this competency-based evaluation strategy.

## Introduction

The College of Family Physicians of Canada (CFPC) Working Group on Postgraduate Curriculum Review recently recommended that each family medicine residency training program in Canada establish a competency-based curriculum in family medicine that is comprehensive, focused on continuity, and centred on family medicine - the Triple C Competency-based Curriculum (Triple C).^1^

Competency-based evaluation entails assessing trainees’ progress in specific outcomes (competencies) on the basis of demonstrated performance.^2^ However, in-training evaluations, largely based on clinical performance ratings throughout the training, are notoriously unreliable, lacking accuracy and reproducibility.^3^ They have shown to be compromised by inadequate performance sampling and inaccurate appraisal due to over-reliance on memory.^4^–^6^

The CFPC Working Group on the Certification Process (WGCP) has made initial recommendations that specifically address how Canadian residency training programs could change their in-training evaluation process to make it more useful for both evaluation and certification purposes. The most far-reaching change the WGCP recommends is that preceptors and residents gather and document case-specific comments and feedback during daily clinical work.^3^ Field notes were developed for this purpose.^7^ Since this tool represents a non-traditional approach that will require changes to the current culture of clinical supervision and evaluation, the WGCP suggested a “try-out” phase of competency documentation during which program directors can see the advantages of field notes but also identify problems and propose solutions.^3^

Evidence suggests that the use of field notes results in more feedback^8^,^9^ as well as better feedback process and structure.^10^,^11^ Furthermore, it helps residents to develop competence through improved performance, learning, reflection, and clinical skills development.^12^ Faculty also seem to believe field notes are an effective teaching, feedback, and reflection tool.^12^ However, little is known about what strategies encourage adoption of their use for documenting competence in clinical teaching practice.

The Theory of Planned Behaviour (TPB) is a social cognitive theory that has been found useful for attempting to predict healthcare professionals’ intentions and behaviours.^13^ While adoption of field notes as a clinical teaching strategy may qualify generally as health professional behaviour,^14^–^17^ there are no previous studies that use the TPB to investigate clinical supervision behaviour specifically in the area of medical education.

**Figure 1:**
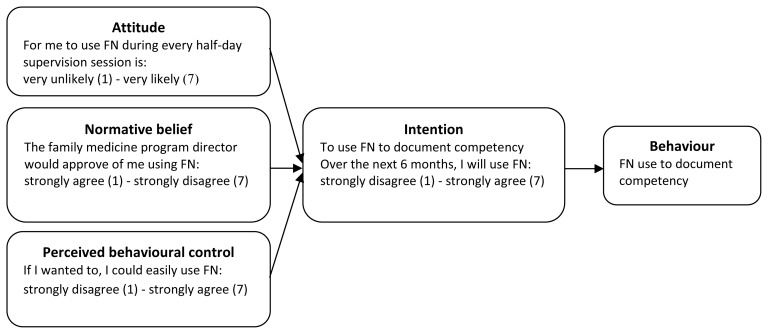
Conceptual framework used – Theory of Planned Behaviour Conceptual framework used for the project, with associated examples of original questions (translated from French) to assess each variable of Ajzen’s Theory of Planned Behaviour. FN = field notes.

The TPB is based on the assumption that actions adopted by human beings are founded on reason,^18^ i.e., people consider the implications of their actions before engaging in a behaviour. According to the TPB, the intention to adopt a given behaviour is the most immediate determinant of the behaviour occurring. As for intention itself, this variable is determined by a) the person’s *attitude*, or degree of affect, favourable or unfavourable, towards adopting the behaviour, b) the *normative belief***,** or their perception of the social pressure to perform the behaviour, and c) the *perceived behavioural control*, or their perception of how easy or difficult it will be to engage in the behaviour (Figure 1). These in turn are influenced by a person’s salient beliefs regarding each factor.^18^

Based on these elements of the TPB, we thus sought to identify the extent to which these factors predict the intention to adopt field notes to document competency in family medicine training, and to elicit the salient beliefs underlying each factor.

## Methods

### Design, setting and participants

**Figure 2:**
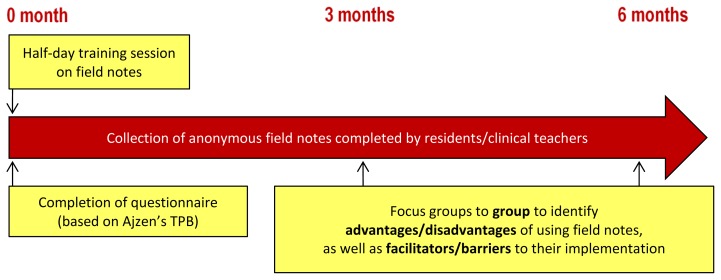
Design Longitudinal design for mixed-methods study to identify predictors for intention to adopt field notes as a competency-based evaluation strategy.

This educational intervention evaluation followed a mixed-methods longitudinal design (Figure 2). The setting for this study was decided following a decision by Université Laval’s Family Medicine Program to adopt field notes as a tool for documenting competency in two volunteer family medicine units in their clinical teaching network. The directors of both units agreed that their teaching sites should participate in the project. The study population therefore included all family medicine residents and clinical teachers in these two units. The eligible participants were those on site during the study period (September 2010 to February 2011).

### Study protocol

In September 2010, a half-day training session on the use of field notes (a one-hour tutorial presentation with a video of a feedback session showing appropriate use of field notes) was delivered to ensure that all participants had a common understanding of the studied behaviour. During the session participants also received a written guide on the use of field notes. Residents and clinical teachers were advised as follows:

- at the beginning of each half-day supervision session: define a competency-based learning objective upon which you would like feedback at the end of the session;
- at the end of the session: discuss a specific competency-oriented feedback and document it as a field note.

### Outcome measures

The main outcome measures in this project were: 1) intention (and its three predictors) to use FN, and 2) salient beliefs, explored in focus groups. Field notes completion rate over the six-month project duration was also measured, as a secondary outcome.

### Quantitative methods (intention and completion rate)

At the beginning of the six-month study period, clinical teachers and residents completed a questionnaire about their intention to implement FN developed in line with Ajzen’s Theory of Planned Behaviour.^18^,^19^ Each variable of the theory (intention and its three predictors: attitude, subjective norm and perceived behavioural control) was assessed with at least three questions with responses based on a 7-item Likert scale (1: Strongly disagree; 2: Disagree; 3: Somewhat disagree; 4: Neutral; 5: Somewhat agree; 6: Agree; 7: Strongly agree). Since we already knew that the three groups of individuals (program director, colleagues and students/supervisors) were likely to act under social pressure to perform the behaviour of using field notes, subjective norm was replaced by its indirect variable, normative belief. Questionnaires for residents and clinical teachers were each approved by two experts. Examples of questions are given in the conceptual framework illustrated in Figure 1. Internal consistency tests showed the questionnaires had good internal validity in both family medicine units (α: 0.66 to 0.90). Thus no items were excluded from the statistical analysis.

During the study period, all completed field notes were collected by the investigators to assess FN completion rate over the six-month project duration. The field notes were identified only with the resident’s and clinical teacher’s respective participant code, to ensure confidentiality.

### Qualitative methods (salient beliefs measure)

Two focus group interviews were conducted in each study setting three and six months after the start of the study period. The residents and clinical teachers met in two focus groups to collect additional comments that might not be raised in resident-only or clinical teacher-only sessions or in the initial questionnaire. Focus groups were audiotaped and subsequently transcribed. Participants were invited to write down any sensitive comments they might wish to record anonymously. Through open-ended questions, the focus groups explored salient beliefs, expressed in terms of perceived advantages/disadvantages and facilitators/barriers.

### Data analysis

Sociodemographic data were presented as means ± standard deviations. Data analysis covered descriptive statistics, internal consistency tests and multiple regression analysis to predict both the intention to use field notes and the actual behaviour of using them.

Quantitative data from the questionnaires were analyzed by computing descriptive statistics of the main constructs of the TPB. Factor analysis and bivariate analysis using Spearman’s correlation coefficients were performed to evaluate the association between each construct and the intention to use field notes. Multivariate analyses were conducted to identify which TPB construct independently explained the most variance in behavioural intention. All statistical analyses were performed using the SAS statistical package (SAS Institute Inc. 2010. SAS OnlineDoc^®^ 9.2. Cary, NC: SAS Institute Inc.). A *p*-value of < 0.05 was interpreted as statistically significant.

Qualitative data from the focus groups were coded using a content analysis procedure. Codes were then sorted into underlying salient beliefs that matched each predictor of intention according to the TPB. We ensured the internal validity of the overall classification by performing an inter-observer reproducibility analysis on the coded data. External validity was ensured by using data matrices to identify the point of saturation, defined as the top 75% most frequently occurring beliefs in the content analysis procedure.

## Results

### Participant characteristics

A total of 27 clinical teachers and 28 residents (18 R1 and 10 R2) participated in the project, for a recruitment rate of 90% and 70% respectively. Table 1 shows the participant characteristics.

### Factors influencing the adoption of field notes

Among clinical teachers, mean intention to use field notes was 6.20 ± 1.20 on a 7-item Likert scale. Residents’ intention was 5.74 ± 1.03. The questionnaire results showing the constructs that directly predict intention are presented in Table 2.

Age, site and status (resident versus clinical teacher) were external factors considered in the analysis (bivariate analysis (age: 0.459; site: 0.009; status: 0.079)). However, even though the teaching site seemed statistically significant for predicting intention, it was not a predictor when added to the model.

**Figure 3:**
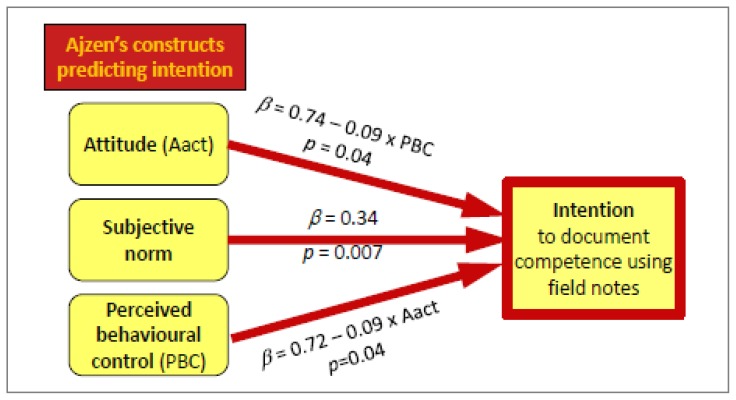
Multiple regression model of the intention to use field notes Multiple regression model of the intention to use field notes. *Attitude (Aact)* and *perceived behavioural control (PBC)* were mutually influential because of an interaction term (*p <* 0.05) and together explain part of the intention to document competence using field notes. The other predictor of intention was *normative belief* (*p <* 0.01). Age, site and status (clinical teacher versus resident) were not predictors of intention when added to the model.

Figure 3 shows how attitude (Aact) (*β*=0.74−0.09 × PBC, *p* < 0.05), perceived behavioural control (*β* = 0.72−0.09 × Aact, *p <* 0.05) and normative belief (*β* = 0.34, *p* < 0.01) act as predictors of the intention to use field notes. The existence of an interaction term between attitude and perceived behavioural control elicits the relationship between these two variables.

### Salient beliefs

Table 3 displays the salient beliefs underlying clinical teachers’ and residents’ attitudes, normative beliefs and perceived behavioural control in regard to documenting competencies using field notes in family medicine training. Data reached saturation for half of the themes.

### Field notes completion rate

Field notes were completed in 31.3 ± 29.0% of supervision sessions (17.4 ± 17.5% of sessions in Site A and 52.8 ± 30.7 % of sessions in Site B). There was no significant correlation between intention and field notes completion rate (*p* = 0.23).

## Discussion

Our results indicate that the family medicine residents and clinical teachers who participated in this project strongly intend to adopt the use of field notes for documenting competence in family medicine training. All three determinants significantly influenced this intention, i.e., attitude, perceived behavioural control (each with modest influence but mutually influential, as demonstrated by an interaction term), as well as normative belief. In response to open-ended questions, focus groups spontaneously identified the advantages/disadvantages, facilitators/barriers, and social pressures that explained these factors. But finally, no significant correlation was found between this strong intention and the field notes completion rates. Consideration of these findings lead us to make four main observations.

First, in conformity with the findings of a systematic review by Godin et al.^13^, this project demonstrates that the Theory of Planned Behaviour and its underlying variables appear to be helpful as a framework for assessing healthcare professionals’ intentions to adopt behaviours, including the use of educational tools. As far as we are aware, this is the first study that has applied the theory to a clinical supervision behaviour in postgraduate medical education.

Second, baseline intention was high because immediately after the training session (at baseline), residents and clinical teachers felt favourably towards competency documentation. This might be explained by an ongoing search on the part of clinical teachers for an organized approach to assessment^7^ and residents’ need for constructive and informative feedback.^20^

Third, the lack of a significant correlation between intention and the field notes completion rate suggests that strong intention does not automatically translate into a high likelihood of implementation. Since low intention-behaviour correlations often reflect a tendency for intentions to overestimate readiness to perform socially desirable behaviours,^21^,^22^ the intention to use field notes was probably overrated by residents and clinical teachers aware of the upcoming accreditation standards. Moreover, the lack of correlation can also be explained by the fact that, although clinical teachers and residents were highly motivated to perform the behaviour and had a generally favourable attitude, they may not have felt that they had enough control over the completion of their field notes,^22^ a process that depends on multiple and coordinated actions by both clinical teachers and residents. Each group’s lack of control over the behaviour of the other might therefore have influenced the completion rate. In addition, although the behaviour was explained prior to implementation using a video to illustrate expectations,^24^ it remains a complex behaviour, which might have also influenced its adoption. The lack of correlation may thus reflect on the usefulness of the unadjusted TPB model itself, which is more efficient in linking intention with behaviour when the behaviour is more specific or easier to apply.^25^

Fourth, the lack of correlation may be related to a lack of agreement as to whether field notes are useful at all. Some of the advantages of field notes identified by the focus groups, such as improved feedback^8^–^10^,^26^ and better integration of resident input into the supervisory process,^20^ were coherent with previous studies on their usefulness. However, Kogan et al.^27^ have shown that despite evidence that students received abundant feedback and in the area(s) they had requested, satisfaction was low. Moreover, their study found the mean rating of feedback usefulness was significantly lower *after* implementation of feedback cards (a tool similar to field notes) than before. The disadvantages of using field notes identified by the focus groups in our study (poor cost-effectiveness and lack of longitudinal representativeness) as well as some barriers (lack of time, vocabulary, and tools for follow-up or progress assessment), may throw some light on the reasons behind Kogan’s observations. It is important to mention that study participants had never used field notes in the past when intention was measured, so they may have discovered disadvantages and barriers after implementation that could not influence their original intention, leading to overrated intention and a lack of significant correlation between intention and behaviour.

In addition, the identified barriers are also consistent with the feedback obtained from Ross et al. after their implementation of a competency-based assessment system using field notes: residents mentioned a general lack of direction, increased workload and lack of preceptor training to deliver specific feedback.^28^ To increase buy-in to field notes, further research should work on limiting disadvantages and decreasing barriers. For example, better cost-effectiveness, quality of feedback and longitudinal representativeness of residents’ competency might be obtained through electronic field notes and portfolio, since our focus groups mentioned that electronic tools for documenting competency would encourage more frequent and detailed feedback, an observation confirmed by other studies.^29^ Ensuring regular follow-up of progress with properly trained faculty advisors^28^ would also help residents to understand individual field note comments in the context of general competency progression, therefore helping to improve longitudinal representativeness. However, the resulting increased workload might only be balanced with improved perception of the usefulness of field notes.

Despite many reasons to explain the lack of correlation between intention and behaviour in our study, measuring the intention and its predictors with the TPB model is still relevant. According to Ajzen’s theory, intention is the main predictor of behaviour.^18^ It was also found to explain 27% of the variance in behaviour in a meta-analysis of 185 studies.^30^ Understanding the factors that influence the intention to adopt field notes will help for their implementation by targeting interventions towards the primary determinants of intention, i.e., the perceived advantages/disadvantages (attitude) and facilitators/barriers (perceived behavioural control) of using field notes.

This project has some limitations. First, we acknowledge that this study uses a small sample size. However, its results will help in planning more effective interventions in a larger number of teaching units. Second, since this project was carried out in two volunteer family medicine units, there was an obvious volunteer bias. However, our team deliberately chose to pilot this project in a favourable setting to give impetus to using the tool in these clinical settings later on. A major strength of this project was the study team, which included clinical teachers as well as junior and senior researchers specialized in both qualitative and quantitative approaches. This collaboration between clinicians and researchers ensured a good balance between the practical issues faced by teaching units and the conceptual approaches upon which the study was based. In our mixed-methods approach, the qualitative data confirmed and enriched our comprehension of the salient beliefs underlying the attitude, perceived behavioural control and normative belief, constructs that had been identified through a quantitative methodology.

In conclusion, the intention to adopt field notes to document competency is influenced by the closely related variables of attitude and perceived behavioural control, as well as by normative belief. Since many clinical teaching settings are not yet ready for this major change in medical education, this study will help family medicine programs to understand the factors that influence the intention to adopt such a competency-based evaluation strategy that is expected to become an accreditation standard in the coming years. Implementation of field notes should be preceded by interventions that target the salient beliefs that influence these primary determinants of intention, i.e., the perceived advantages/disadvantages (attitude) and facilitators/barriers (perceived behavioural control) of using field notes, in order to improve this evaluation strategy. Clear expectations on the part of programs (normative belief) should also improve adhesion.

## Figures and Tables

**Table 1:** Participant characteristics

		Clinical teachers	Residents
		
**Age (yrs)**		42.3±10.7	29.2±6.1
**Training level**	**R1 (*n*)**		18
	**R2 (*n*)**		10
**Teaching experience (yrs)**		6.6±8.3	
**Site**	**A**	12	17
	**B**	15	11

**Table 2:** Questionnaire results on direct constructs predicting intention

	Clinical teachers	Residents
**Intention**	6.20±1.20	5.74±1.03
**Attitude**	5.56±1.22	4.93±1.47
**Normative belief**	6.19±0.88	5.89±0.99
**Perceived behavioural control**	**5.47±1.42**	**5.15±1.34**

**Table 3:** Focus group results: salient beliefs

Constructs	Salient beliefs discussed in focus groups	
Behavioural beliefs	Advantages Facilitates feedback Promotes residents’ autonomy/self-assessment, more active in their supervision	Disadvantages Poor cost-effectiveness/time-consuming Daily feedback only; lack of longitudinal representativeness (too specific/punctual)
Control beliefs	Facilitators Some teaching contexts: emergency room setting, daytime supervision/direct supervision Protected time Electronic field notes Additional training for faculty advisors (faculty development)	Barriers Overwhelming Identification of a learning objective at the beginning of half-day Lack of time Lack of vocabulary/”blank sheet syndrome” Unclear goal/usefulness Lack of tools for follow-up and assessment of competency progression
Normative beliefs	People who would approve or disapprove of using field notes Family medicine program (if clear expectations) Teachers (easier in a culture of daily feedback)	
